# Perceiving What Is Reachable Depends on Motor Representations: Evidence from a Transcranial Magnetic Stimulation Study

**DOI:** 10.1371/journal.pone.0002862

**Published:** 2008-08-06

**Authors:** Yann Coello, Angela Bartolo, Bastien Amiri, Hervé Devanne, Elise Houdayer, Philippe Derambure

**Affiliations:** 1 Lab. URECA (EA 1059), Université de Lille–Nord de France, Lille, France; 2 UMR-CNRS 8163 STL, Université de Lille–Nord de France, Lille, France; 3 Département de Neurophysiologie Clinique, Hôpital Roger Salengro, Centre Hospitalier Régional Universitaire, Lille, France; Harvard Medical School, United States of America

## Abstract

**Background:**

Visually determining what is reachable in peripersonal space requires information about the egocentric location of objects but also information about the possibilities of action with the body, which are context dependent. The aim of the present study was to test the role of motor representations in the visual perception of peripersonal space.

**Methodology:**

Seven healthy participants underwent a TMS study while performing a right-left decision (control) task or perceptually judging whether a visual target was reachable or not with their right hand. An actual grasping movement task was also included. Single pulse TMS was delivered 80% of the trials on the left motor and premotor cortex and on a control site (the temporo-occipital area), at 90% of the resting motor threshold and at different SOA conditions (50ms, 100ms, 200ms or 300ms).

**Principal Findings:**

Results showed a facilitation effect of the TMS on reaction times in all tasks, whatever the site stimulated and until 200ms after stimulus presentation. However, the facilitation effect was on average 34ms lower when stimulating the motor cortex in the perceptual judgement task, especially for stimuli located at the boundary of peripersonal space.

**Conclusion:**

This study provides the first evidence that brain motor area participate in the visual determination of what is reachable. We discuss how motor representations may feed the perceptual system with information about possible interactions with nearby objects and thus may contribute to the perception of the boundary of peripersonal space.

## Introduction

The conscious experience of a continuous external world contrasts to some extent with the necessity to represent a discontinuous action space: What we see is not always what we can reach. More specifically, although the world in which one moves is experienced as homogenous and continuous both through time and space, interactions with objects available in our surrounding space necessary depends on body properties. A glass can be grasped only if our arm is long enough to reach it, and only if our fingers are strong enough to lift it. Hence, near-peripersonal space must be differentiated from far-extrapersonal space, and this ability obviously depends on our past experiences about opportunities, consequences and costs of acting in the environment with our own body [1]. In these terms, peripersonal space is defined as the space immediately surrounding our body. Objects within peripersonal space can be grasped and manipulated; objects located beyond this space (extrapersonal space) cannot normally be reached without moving towards them. This suggests that the brain should represent objects situated in peripersonal space differently from those in extrapersonal space [2]–[4].

Though it has long been suspected that the visual perception of objects within and out arm's reach may be subserved by different brain mechanisms [5], [6], it is only recently that a small number of studies have investigated the neural basis of near-far dimensions. In the context of object recognition, it has been suggested that the dorsal visual stream is primarily implicated in attending to objects in near space, whereas the ventral visual stream is primarily implicated in attending to objects in far space [2], [7], [8], which fits quite well with the dual visual system hypothesis [9]. In the same vein, clinical dissociations between attending to objects in near and far space have been reported. Studies of radial line bisection performed within arm's reach (peripersonal space) have shown that bilateral temporo-occipital lesions can be associated with a significant misbisection towards the body, interpreted as neglect of far space [10]. By contrast, lesions of bilateral parieto-occipital cortex have been associated with a significant misbisection away from the body, interpreted as neglect of near space [11]. These data suggest a dissociation in perceiving objects in the upper and lower visual field according to gaze direction. In a different context, Bjoertomt, Cowey and Walsh [12] also probed the involvement of the ventral and dorsal stream in processing near and far space during a horizontal line bisection task, but using repetitive transcranial magnetic stimulation. The subjects' task was to indicate whether the part of the line to the left or right of the transection appeared longer. Results showed that the magnetic stimulation of the right posterior parietal cortex and the right ventral occipital lobe selectively induced a significant shift to the right in the perceived midpoint for near- and far-space lines, respectively. According to the authors, this dissociation supports the hypothesis of a dorsal/near space-ventral/far space segregation of processing within the visual system. However, space representation for object recognition is different from space representation for action [9], [13]. To interact with an object, it is necessary to determine whether this object is in the near-reachable space or in the far-not reachable space. Although perception for action is thought to mainly involve the dorsal stream of the visual system [9], how the transition from near to far space is specified within the brain in the context of action remains an open issue. Recently, it has been proposed that the transition might be gradual, with no abrupt shift at arm's length [14], but this still does not provide any assumption about how the transition can be specified at a neural level.

In the past, several studies have suggested that people are quite accurate in visually determining the boundary of the reachable space. Classically, the critical test consisted in placing individuals facing a horizontal surface and to present series of visual objects in increasingly near and far locations along the sagittal axis. In this context, the participants' task was simply to provide an overt verbal response about whether the visual object was reachable or not with their hand. In such judgement task, no actual movement was performed and the mobility of the trunk was generally restricted. When using this method, the general agreement was that what is reachable with the hand depends mainly on the distance of the target relative to the length of the arm [15]–[19]. Thus, determining whether a visual object is reachable or not is essentially a function of the observer's perceived body-capabilities, which generally slightly overestimates the true arm length [15], [17], [18]. Such an overestimation was interpreted as originating from people's everyday experience of reaching, which naturally requires multiple skeletal degrees of freedom, whereas they are generally tested in restricted postural situations that prevent natural body movements [17]–[19]. In agreement with this interpretation, when evaluating the boundary of the reachable space without postural constraints, i.e., using the torso and the arm instead of merely the arm, the overestimation significantly diminished [15], [19], but it nevertheless persisted. Alternatively, it is acknowledged that overestimations can also have a perceptual origin [20]. In many of the experiments about judging what is reachable, participants were required to provide a perceptual judgement for visual objects presented in a dark visual scene. The structure of the visual scene is known to have an overall influence on the distance at which visual objects are perceived [21], [22] and we recently reported that when reachability judgements are performed according to stimuli presented on a textured rather than an homogeneous dark surface, overestimation reduces significantly and the boundary of what is reachable becomes very close to actual arm length [20].

Because body postural control and limb movements are context dependent, we may suppose that the capacity to perceptually discriminate what is reachable from what is not reachable involves not only information about properties of objects as revealed by scene-based and gaze-related visual inputs, but also information relating to the body and the possibilities of acting with it. Recently, Coello and Delevoye-Turrell [23] reported the case of a patient, G.L., who suffered from a peripheral deafferentation and had great difficulty in performing a reachability judgement task while she was still able to perform accurate reaching movements and had no visual impairments. The authors interpreted this striking result by suggesting that reachability judgements in healthy persons may depend on visual information formatted by implicit knowledge about expected sensory consequences of potential motor actions. This interpretation refers to the well-known control theory framework [24], [25]. According to this theory, a visual stimulus located in the proximal space can automatically evoke a “potential motor action” which, regardless of whether the action is subsequently executed or not, maps the spatial stimulus position in motor terms [26]. By generating a covert action through an internal model, the motor system can simulate a motor command in relation with a particular visual stimulus (the inverse model) and can predict and anticipate the sensory consequences of the action through a predictive model (the forward model) [24], [27], [28]. The crucial aspect of the theory is that the function of the whole simulation-prediction process, that may include the well-known mirror neuron system, would be not only to make the motor system ready for action and more efficient for on-line control during execution, but also to provide the agent with information about the feasibility of potential actions. Thus, internal signals associated with covert motor activity would enable an estimation of body capabilities, which could subsequently be used for the determination of peripersonal space [23].

Motor representations are thought to involve a neural network that overlaps with the one activated during motor planning and motor execution, and also during motor imagery and even motor cognition [29]. It has been shown that this is particularly true for the motor and premotor cortices, the supplementary motor area and the posterior parietal cortex [30]–[33]. Whether this network is also involved in the perceptual judgement of what is reachable represents the aim of the present study. One way to test the involvement of motor representations in the perception of what is reachable consists in applying a transient perturbation in the form of a TMS pulse over the motor areas while performing the perceptual task. TMS at a frequency equal to or below 1 Hz has the effect of depressing cortical excitability for a short period of time after each pulse [34]. Thus, TMS introduces noise into the system being stimulated and it can therefore be employed as a technique producing transient virtual lesion. According to the control theory, applying a TMS pulse on the motor brain areas should perturb the perceptual judgement of what is reachable spatially and/or temporally. However, Schluter et al. [35] found a different effect of TMS on the premotor and motor areas suggesting differentiated network involved in movement selection and execution. In particular they found that premotor cortex stimulation alone disrupts an early stage of movement selection, whereas motor cortex stimulation disrupts the movements at a later stage of execution. On the basis of these data, we decided to stimulate three different cortical sites at a subthreshold intensity: the left motor area associated with the right radial extensor carpi activation (involved in grasping movement), the left premotor cortex and the left temporo-occipital area used as control site, while perceptually judging what is reachable with the right hand. A perceptual right-left decision (control) task and an actual grasping movement task were also administered. Since the effect of transcranial magnetic stimulation could occur at different stimulus onset asynchronies (SOA) after target presentation [35], [36], TMS pulse was delivered 50ms, 100ms, 200ms or 300ms after target presentation and performances were compared to the condition with no TMS. Previous studies have reported longest reaction times for motor response when TMS was delivered above threshold intensity, whereas shortest reaction times occurred to TMS at subthreshold intensity [37], [38]. Moreover, increasing SOA between the stimulus presentation and the TMS has for consequence that reaction time is progressively delayed [39]. Furthermore, multisensory stimuli (e.g. a combination of visual, auditory and tactile information) determine an inter-sensory facilitation improving time processing, as this was the case with increasing intensity of the stimulus signal [37], [40]. We can thus expect that stimulating the motor areas should reduce reaction times, but this effect would depend on the SOA. Moreover, TMS pulse on the motor brain areas should disturb the perceptual judgement of what is reachable spatially and/or temporally, whereas other discrimination tasks such as the right-left decision task should remain unaffected. Finally, because brain stimulation did not usually show any effect on the pattern of the agonist/antagonist muscle activities and the overall form of the movement when providing a motor response [37], we expected no effects of the various stimulation conditions on the kinematics of the grasping task.

## Methods

### Participants

Seven right-handed volunteers ([41]: laterality quotient 86% on average, SD: 10%, ranging from 72% to 100%), from the University of Lille3 with no history of neurological or psychiatric illness took part in the experiment (4 females and 3 males, mean age 25.4 years). All participants had normal or corrected-to-normal visual acuity and were naïve as to the purpose of the experiment. They all gave their informed consent prior to their inclusion in the experiment, which was approved by the University Charles de Gaulle and University hospital ethics committees and in accordance with the principles of the Helsinki 1964 declaration.

### Apparatus and procedure

**Figure 1:**
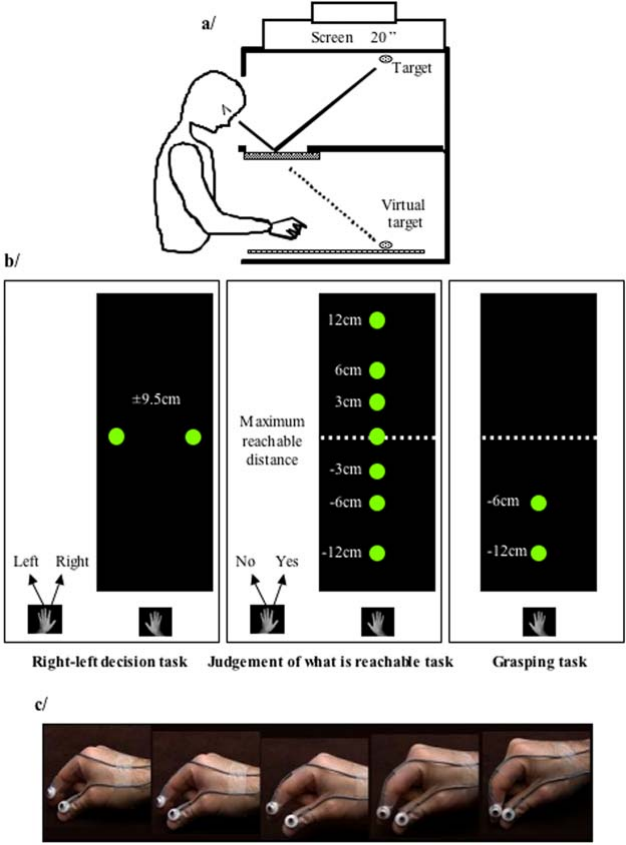
A schematic representation of the experimental apparatus and target display in the right-left decision task, the judgement of what is reachable task and the grasping task. (a) When looking into the apparatus, the bottom part is visible only through optical projection upon the mirror of information coming from the upper part of the apparatus. (b) Targets display in the right-left decision task, the judgement of what is reachable task and the grasping task. (c) Kinogramme of actual grasping movement.

The experimental apparatus consisted in a rectangular box (60cm high, 80cm wide and 80cm deep) divided horizontally by a half-silvered mirror (see Figure 1a). A 19 inches flat panel computer monitor (Dell1907FP) was placed upside-down on the top surface of the apparatus so that the image generated by the computer was reflected in the mirror. Due to the optical geometry, the image on the computer screen appeared to be projected onto the bottom surface of the workspace. The visual stimulus was represented by a white 2-D dot (1.5cm diameter) displayed during 3 seconds on a dark background. The inter-stimuli interval (ISI) was 4 seconds. The inner surfaces of the box were smooth and painted matt black. No visual information from the external environment was available during the entire experiment. All subjects underwent two perceptual tasks: a right-left decision task and a reachability judgement task. In the right-left decision task (30 trials), which served as a control task since this perceptual task was without motor content, two targets located laterally at ±9.5cm from sagittal axis and about 60cm from the subject's body were randomly presented and the subjects' task was to respond by lifting the left index finger when presented with the right dot and the left middle finger when presented with the left dot (half of the group had the inverse attribution of the fingers). In the reachability judgement task (420 trials), 7 dots were displayed along the radial axis at different distances according to maximum arm length (0cm, ±3cm, ±6cm, ±12cm, see Figure 1b). Subjects were required to judge perceptually whether the randomly presented dot was reachable or not reachable. Responses were provided by lifting the left index when the target was judged as reachable or left middle finger when judged as not reachable (half of the group had the inverse attribution of the fingers). Before administrating the perceptual tasks (random attribution), the participants were instructed to perform 30 grasping movements towards a target that was randomly presented at -12cm or -6cm from maximum arm length along the radial axis. Target locations were chosen so that they could easily be reached though accounting for significant variability along the sagittal axis. Furthermore, the location of the targets prevented subjects to get sensorimotor experience associated with the boundary of their reachable space. The movements were performed without direct visual control due to the mirror and ended when the right index and middle fingers were in contact with the virtual object (see Figure 1c for an example of such movement). No feedback about performance was provided to the participants. The hand displacement required an extension of the shoulder, the elbow and the wrist to grasp the target, but since the target was a 2-D stimulus, no actual contact between the fingers and the target was experienced. This task was mainly to familiarise the subjects with the type of motor performance that was involved in the reachability judgement task.

### Transcranial magnetic stimulation

Magnetic stimulations were delivered using a 9.5 cm external diameter figure-of-8 focal coil connected to a Magstim 200 stimulator (The Magstim Company Ltd, Whitland, UK). Stimulation was applied over the optimal scalp point for the Extensor Carpi Radialis (ECR), i.e. the site which yielded the strongest ECR motor evoked potentials (MEPs) at a given suprathreshold intensity. This muscle is an extensor of the wrist joint, and travels along the radial side of the arm. It enables movement of the hand and the wrist, and is recruited when reaching for objects along the radial axis. Moreover, previous studies have shown that for reaching movements, activations of shoulder, elbow and wrist muscles involve common motor cortical circuits [42] and these muscles have overlapping motor cortical representations [43]. The ECR muscle seemed thus to be the most appropriate one. Indeed, in the rest condition, subjects had their right arm lying on the table, folded against their chest, palms down. Consequently, when they reached for the target, one of the first movements they had to perform was to extend their wrist in the same time as the extension of the arm towards the target. EMG signals were constantly monitored in each subject, to ensure that each movement was accompanied by an ECR contraction, from the beginning to the end of the movement. This muscle is thus thought to be involved at a subthreshold level for the generation of covert actions when required to perform a reachability judgement task. The ECR's optimal scalp point was determined by moving the coil over the hand motor area while the subjects relaxed their arm muscles. In order to ensure constant coil positioning throughout the series, the ECR's optimal scalp point was marked on a swimming cap worn by the subject and the coil was held in place by a mechanical device. The coil was held tangentially to the scalp, with the handle pointing backwards and laterally (at a 45° angle from the midline). We then measured the ECR's resting motor theshold (RMT), defined as the lowest possible stimulus intensity capable of inducing MEPs greater than 50 μV in at least 5 out of 10 trials. For the three experiments, stimulation was applied at 90% of the RMT. This corresponded on average to 56% of the maximum intensity of the stimulator. Bipolar Ag-AgCl surface electrodes were used to record the electromyographic (EMG) activity of the ECR. EMG signals were amplified (×1000), high-passed at 10 Hz and low-passed at 1000 Hz (Grass Technologies, West Warwick, USA) prior to sampling at 2 kHz with a 1401MicroMKII device (Cambridge Electronic Design, Cambridge, UK).

Considering the stimulated sites, the location for the left motor area was defined from the motor evoked potentials (MEPs) obtained in the contralateral arm on the ECR. To locate the left premotor site, we referred to Schluter et al. [35], and placed the coil 2cm anterior and 1cm medial to the motor site. As in previous TMS studies on motor control [44], [45], we chose a left temporo-occipital site as a control site and the location for the TMS was selected by placing the coil halfway between a line from the inion to a point 7 cm lateral to the motor area [46], [47]]. The brain sites were stimulated in a counterbalanced order. The spatial resolution of the TMS was supposed to be in the order of a few millimetres [48] and single pulse stimulation was supposed to affect brain activity for 15–50 milliseconds. [49].

For each site, the subjects received single subthreshold TMS pulses with onset asynchronies of 50ms, 100ms, 200ms, and 300ms after stimulus presentation [36]. TMS stimulations at different SOAs were alternated with a no-stimulation condition in a pseudo-random order. In the right-left decision task, subjects received for each site (motor, premotor and control site) 24 stimulations (3 trials×2 targets×4 SOAs), and for 6 trials they received no stimulation. In the reachability judgement task, subjects received for each site 112 stimulations (4 trials×7 targets×4 SOAs), and for 28 trials they received no stimulation. Participants were also required to perform actual grasping movements and they received for each site 24 stimulations (3 trials×2 targets×4 SOAs), and for 6 trials they received no stimulation while executing the movement. Overall, each subject received 480 stimulations during the experimental session.

### Magnetic resonance imaging (MRI)

**Figure 2:**
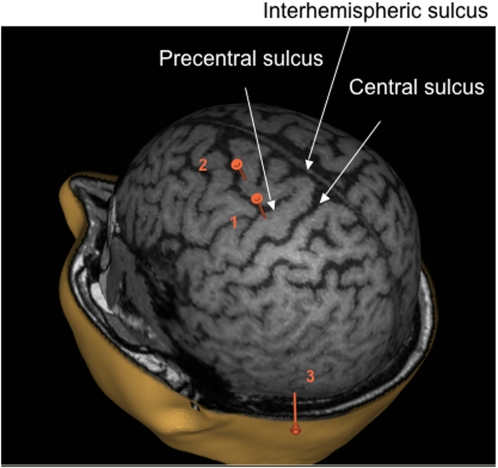
MRI reconstruction of the three-dimensional brain volume for one subject. Three sites were targeted : (1) the primary hand motor area, (2) the premotor area and (3) a control site lying in the temporo-occipital region.

On a separate occasion and for illustrative purposes, a high-resolution anatomical MRI of one subject was obtained. Images of brain anatomy were determined with a series of high-resolution MRI scans. Scans were acquired on a 1.5-Tesla Philips Gyroscan NT scanner. A T-1 weighted, three dimensional, fast-field echo pulse sequence of 160 contiguous 1.3mm coronal sections was obtained (TR, 33 ms; TE, 12 ms; flip angle, 35 degrees). A three-dimensional representation of the cerebral cortex was computed using the eXimia Navigated Brain Stimulation system (NexStim Ltd, Helsinki, Finland). The subject's head position was monitored in real-time using a Polaris Optical Tracking system. A pointer was used to target the three cortical stimulation sites. The pointer positions were registered according to the subject's head reference frame and were superimposed onto the reconstructed three-dimensional image of the cortex using the eXimia software (NexStim Ltd, Helsinki, Finland, see Figure 2).

### Behavioural measurements

In the perceptual and the grasping tasks, ultra-sound markers (CMS10 Measuring System from Zebris Medical GmbH Company) were fastened to the left index and middle fingers and to the right thumb and index finger. The markers on the left hand were used to register and analyse subjects' responses in the two perceptual tasks. The markers on the right hand were used to register and analyse the actual grasping performances. In the right-left decision task and the reachability judgement task, temporal performance and response accuracy were analysed from left index and middle fingers movements, those fingers being used to provide the yes-no or reachable-not reachable responses. In all tasks, onset and offset of the finger movements were defined as the time at which velocity exceeded 5 cm/sec for more than 100ms. Reaction time corresponded to the time elapsed between target presentation and the first detected finger movement. Data for the different target positions were initially pooled when analysing the effect of TMS SOAs and the site stimulated. Target by target analysis was performed afterwards in the reachability judgement task. In this task, the boundary of what is reachable was determined using a maximum likelihood fit procedure based on the second-order derivatives (quasi-newton method) to obtain the logit regression model that best fitted the (yes-no) responses of the subject for the seven distances of the target according to arm length (0cm, ±3cm, ±6cm, ±12cm). The logistic function is represented by the following equation:
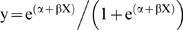
where y is the subject's response, x is the target location, (−α / β) is the critical value of x at which point the transition from one type of response (reachable) to the other type of response (not reachable) occurs thus expressing the mean location of the boundary of peripersonal space, and (β/4) is a measure of the slope at point −α / β.

In the grasping task, kinematic characteristics of hand displacement were analysed in order to obtain information about movement time (MT), peak velocity (PV), peak acceleration (PA) and peak deceleration (PD). Average trajectories across the subjects were also analysed in the various experimental conditions. For statistical investigations, performances were analysed in each task through a two-way analysis of variance (ANOVA: site of stimulation (3)×TMS-SOA (5)), with repeated measures on all factors. When the sphericity assumption was violated (i.e. Epsilon smaller than 1), Huyn-Feldt adjustments of the p-values were reported. Simple effects were used to investigate significant interactions and standard t-test was used for local comparisons.

## Results

### Right-left decision task

#### Reaction time

**Figure 3:**
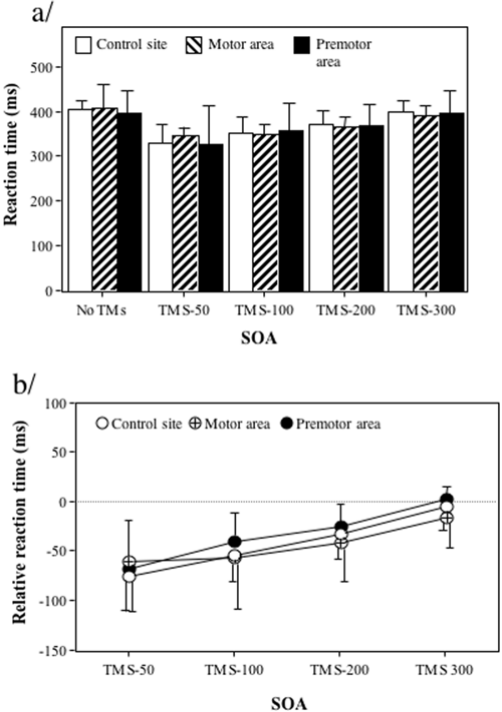
Effect of the SOA and the site where TMS was delivered on reaction times in the right-left decision task. (a) Data represent absolute reaction times (ms) and standard deviations in for the different SOA conditions (no TMS, 50ms, 100ms, 200ms, 300ms) and the different sites stimulated (occipito-temporal complex, motor cortex, premotor cortex). (b). Data represent relative reaction times (ms) and standard deviations according to the no-TMS condition in the different conditions.

Reaction times were on average 372ms and did not differ significantly in the no-TMS condition whatever the brain site condition considered (control site: 406ms, motor area: 409ms, premotor area: 398ms, F(2,12) = 0.21, p = .81). Analysing relative reaction times in relation to the no-TMS condition showed that the effect of the TMS was mainly a decrease in reaction time, which was not significantly different for the different sites (F(2,12) = 0.27, p = 0.72, with control site: -42ms, motor area: -44ms, premotor area: -33ms see Figure 3). Reaction times were however influenced by the SOA (F(3,18) = 16.52, p<0.01). Indeed, a progressive decrease in the relative reaction time was observed from SOA-50ms up to SOA-300ms (with SOA-50ms: -68ms, SOA-100ms: -50ms, SOA-200ms: -34ms and SOA-300ms: -7ms, all values being significantly lower than that observed at SOA-300ms, respectively t(18) = 6.76, t(18) = 4.81, t(18) = 2.98, all p<0.01). There were no interaction between the two factors (F(6,36) = 0.61, p = 0.71). Thus, the main effect of TMS was a facilitation effect, which progressively decreased from SOA-50ms up to SOA-300ms for which it was absent (mean relative reaction time (-7ms) was not different from 0, t(6) = -0.70, p = 0.51).

#### Spatial accuracy

The percentage of errors was 3.7% on average and there were no variations in the directional errors in function of the stimulated site (F(2,12) = 3.67; p = 0.06, with for the control site: 6.19%, the motor area: 2.92% and the premotor area: 1.91%). Directional error was not influenced by the SOA (F(4,24) = 1.50; p = 0.23, with for no-TMS: 0.95%, SOA-50ms: 4.76%, SOA-100ms: 7.14%, SOA-200ms: 3.2%, SOA-300ms: 2.38%) and no interaction between the two factors reached significance (F(8,48) = 2.01; p = 0.14).

### Actual grasping movement

#### Reaction time

**Figure 4:**
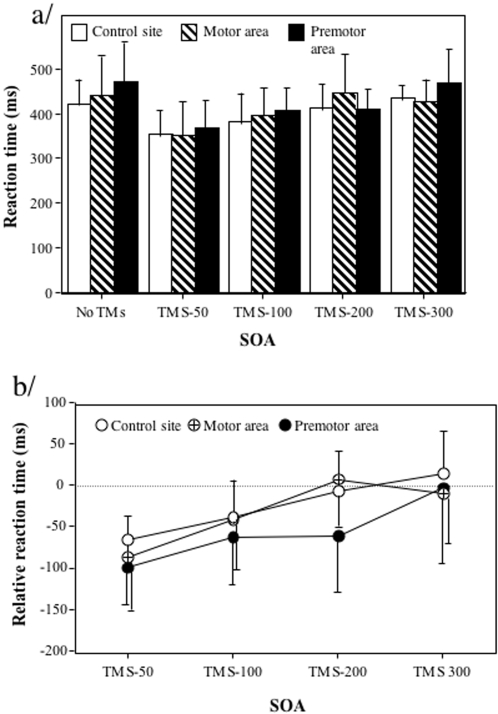
Effect of the SOA and the site where TMS was delivered on reaction times in the grasping task. (a) Data represent absolute reaction times (ms) and standard deviations in for the different SOA conditions (no TMS, 50ms, 100ms, 200ms, 300ms) and the different sites stimulated (occipito-temporal complex, motor cortex, premotor cortex). (b). Data represent relative reaction times (ms) and standard deviations according to the no-TMS condition in the different conditions.

When performing actual grasping movement, reaction times were on average 401ms and did not differ significantly in the no-TMS condition whatever the considered brain site (control site: 409ms, motor area: 427ms, premotor area: 458ms, F(2,12) = 3.74, p = 0.73, see Figure 4). Analysing relative reaction times in relation to the no-TMS condition showed that the effect of TMS was mainly a decrease in reaction times that was very similar when considering the different sites (control site: -25ms, motor area: -33ms, premotor area: -58ms F(2,12) = 1.59, p = 0.24). There was however an effect of the TMS in function of the SOA (F(3,18) = 10.35, p<0.01, with SOA-50ms: -85ms, SOA-100ms: -48ms, SOA-200ms:-21ms and SOA-300ms: -0.2ms). Relative reaction times decreased progressively from SOA-50ms up to SOA-200ms for which they were close to the no-TMS condition (all values being significantly lower than that observed at SOA-300ms except for SOA-200ms, respectively t(18) = 5.27, t(18) = 3.01, t(18) = 1.33, only the two first p<0.01). We observed no interaction between the two factors (F(6,36) = 1.44, p = 0.33). The main effect of the TMS was thus a facilitation effect, which diminished progressively from SOA-50ms up to SOA-200ms for which it was absent (mean relative reaction time (-21.48ms) was not different from 0, t(6) = −0.93, p = 0.33).

#### Movement duration

Movement duration was on average 607ms and was not influenced by the site of stimulation (F(2,12) = 0.83, p = 0.57, with control site: 581ms, premotor area: 620ms and motor area: 619ms). Movement duration was not dependent on the SOA (F(4,24) = 0.19, p = 0.93) and there was no interaction between the two factors (F(8,48) = 0.80, p = 0.56).

#### Kinematic characteristic

**Figure 5:**
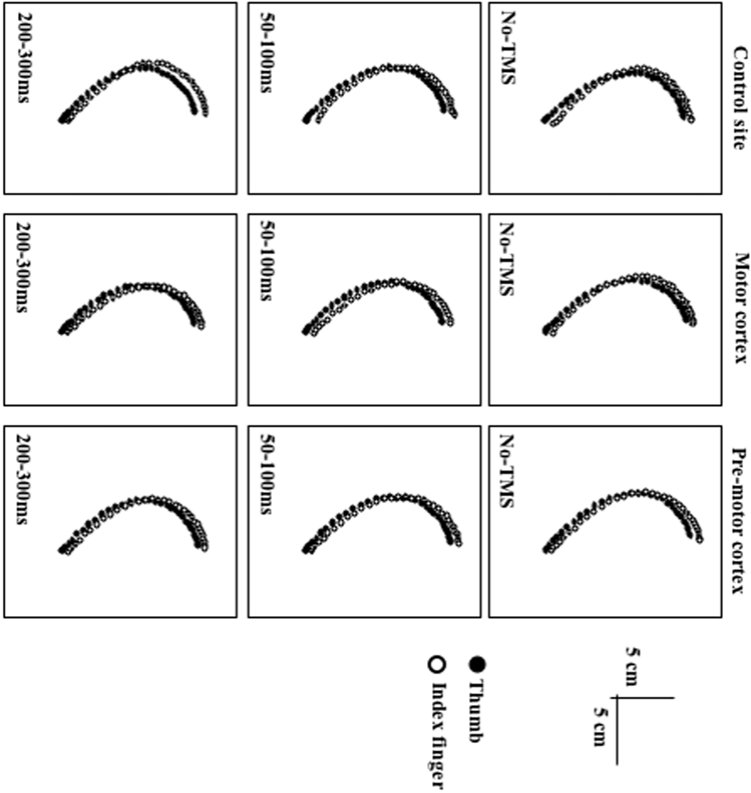
Average trajectories for the thumb and index fingers. Thumb (black circles) and index finger (white circle) trajectories are plotted every 10ms according to the site stimulated (occipito-temporal complex, motor cortex, premotor cortex) in the No-TMS, 50ms-100ms pooled and 200ms–300ms pooled conditions.

**Figure 6:**
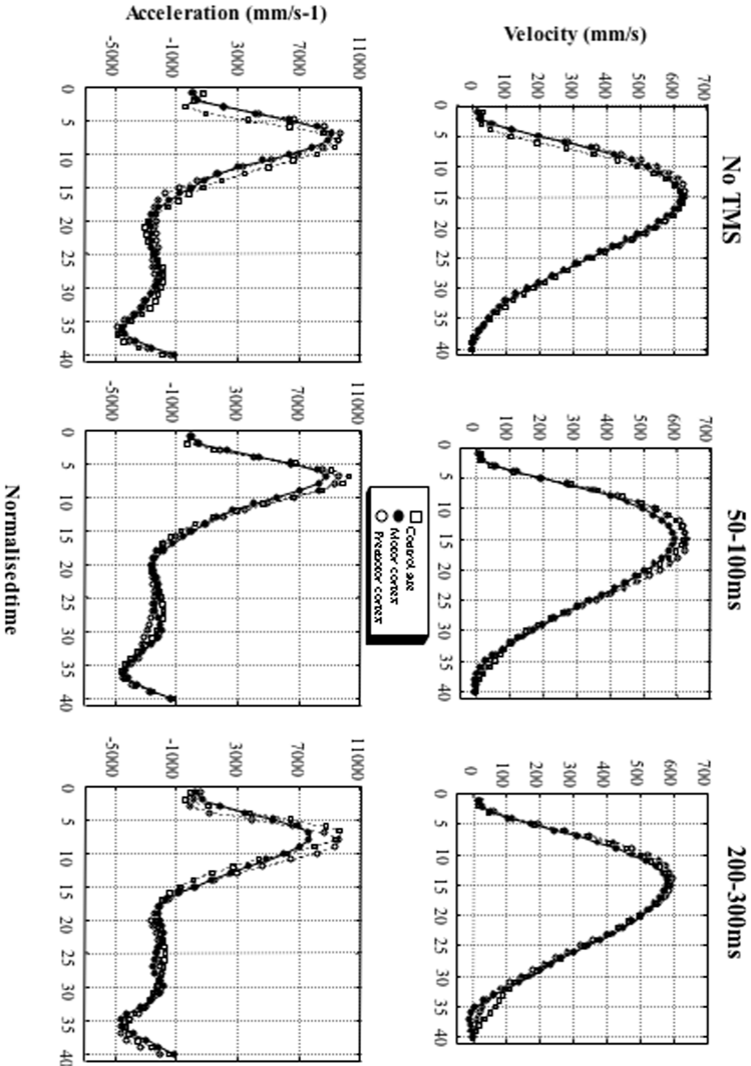
Kinematic analysis of thumb trajectory. Data represent the velocity and acceleration of the thumb as a function of the SOA (No-TMS, 50ms–100ms pooled and 200ms–300ms pooled conditions) and for the different sites stimulated (occipito-temporal complex, motor cortex, premotor cortex).

Mean trajectories and kinematics of the thumb and index finger during virtual grasping movements are shown Figure 5 and 6 as a function of the experimental conditions. Because of the small amount of movements performed in each condition, the trials with a TMS at SOA of 50ms and 100ms were pooled, as well as the trials with a TMS at SOA of 200ms and 300ms. Even though the trajectory amplitude seems slightly shorter when stimulating the motor cortex (11.73cm) than when stimulating either the premotor cortex (12.27cm) or the control site (11.88cm), with concomitant shorter peak velocity (610mm/s, 652mm/s and 640mm/s respectively) and acceleration (8576mm/s^2^, 9485mm/s^2^ and 9649mm/s^2^ respectively), these variations did not reach statistical significance (peak velocity: F(2,12) = 0.92, p = 0.39; peak acceleration: F(2,12) = 0.01, p = 0.97; peak deceleration: F(2,12) = 0.07, p = 0.84) or the SOA (peak velocity: F(2,12) = 0.18, p = 0.76; peak acceleration: F(2,12) = 0.96, p = 0.32; peak deceleration: F(2,12) = 2.27, p = 0.10). No interaction between the factors was observed. Consequently, the TMS did not affect motor execution and this was shown to be true whatever the stimulated site and the SOA.

### Reachability judgement task

#### Reaction time

**Figure 7:**
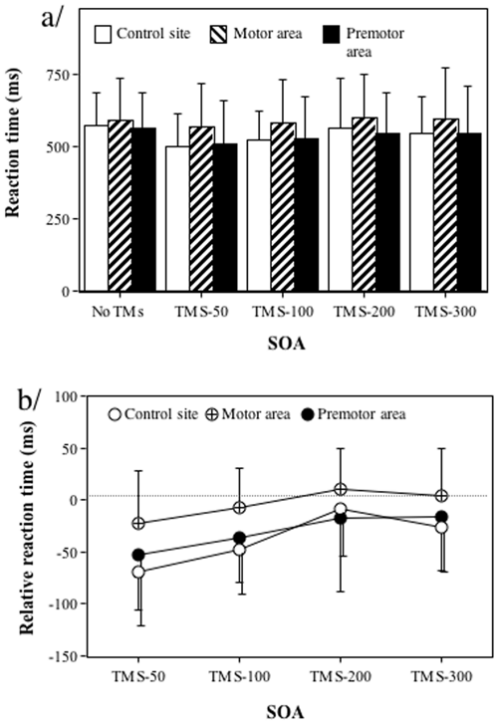
Effect of the SOA and the site where TMS was delivered on reaction times in the perceptual judgement of what is reachable task. (a) Data represent absolute reaction times (ms) and standard deviations in for the different SOA conditions (no TMS, 50ms, 100ms, 200ms, 300ms) and the different sites stimulated (occipito-temporal complex, motor cortex, premotor cortex). (b). Data represent relative reaction times (ms) and standard deviations according to the no-TMS condition in the different conditions.

Reaction times were on average 554ms and, as expected, they did not differ significantly in the no-TMS condition whatever the brain site (control site: 570ms, motor area: 589ms, premotor area: 561ms, F(2,12) = 1.21, p = 0.33, see Figure 7). The analysis of relative reaction times in relation to the no-TMS condition showed that the TMS effect varied significantly according to the stimulated site (F(2,12) = 5.36, p = 0.02). A smaller effect of the TMS was found when stimulating the motor area (-4ms) than when stimulating the two other areas (control: -38ms, premotor site -31ms, t(12) = 3.11, p<0.01 and t(12) = 2.43, p = 0.03 respectively; the two latter conditions being not different, t(12) = 0.69, p = 0.51). Reaction times were affected by the TMS SOA (F(3,18) = 4.57, p = 0.01 with SOA-50ms: -48ms, SOA-100ms: -31ms, SOA-200ms:-5ms and SOA-300ms: -13ms). A decrease of relative reaction times was observed until the SOA-200ms condition for which it disappeared (all values being significantly lower than that at SOA-300ms except for SOA-200ms, t(18) = 2.78, t(18) = 2.09 and t(18) = 0.59, respectively, with only the two first p<0.05). At SOA-200ms, mean relative reaction time (-5ms) was not different from 0, t(6) = −0.26, p = 0.81). There was no interaction between the two factors (F(6,36) = 0.29, p = 0.83) since the weaker effect of the TMS when stimulating the motor area was nearly constant across the SOAs when compared to the premotor and control sites pooled together (SOA-50ms: 38ms, SOA-100ms: 35ms, SOA-200ms: 24ms, SOA-300ms: 24ms). Thus, a facilitation effect on reaction time was observed after cortical stimulation. However, this facilitation was greater when stimulating the control site and the premotor area than when stimulating the motor area, whatever the SOA.

**Figure 8:**
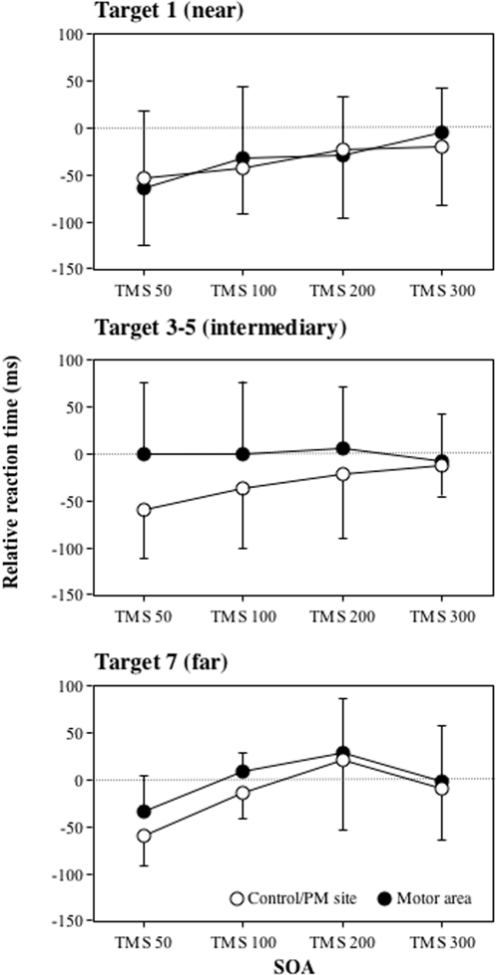
Effect of the SOA and the site where TMS was delivered on reaction times in the perceptual judgement of what is reachable task as a function of target location. Data represent relative reaction times (ms) and standard deviations according to the no-TMS condition for the different SOAs (50ms, 100ms, 200ms, 300ms) and the site where TMS was delivered (motor cortex, contol/premotor area pooled). Upper row: target 1 (near), middle row: target 3 to 5 (intermediary), lower row target 7 (far).

When plotting reaction times against the location of the target for the different SOAs, we found that delivering TMS pulse on the motor cortex induced a similar facilitation effect than with the other sites for the very near (N° 1) and very far (N°7) targets only. No such facilitation effect was found for the targets near the boundary of what is reachable (N° 3–5). Since premotor and control sites provided similar results, data for these two sites were pooled for statistical investigation (see Figure 8). For the very near target, average relative reaction times in function of the no-TMS condition was −36ms and was greater in the SOA-50ms condition (-59ms) tan in the SOA-300ms condition (-12ms, F(1,6) = 6.12, p = 0.04). This effect was similar for all stimulated sites (premotor/control areas: -37ms, motor area: -35ms, F(1,6) = 0.01, p = 0.94) and no interaction between the two factors was registered (F(1,6) = 2.01, p = 0.21). For the very far target, relative reaction times in function of the no-TMS condition was -26ms and was greater in the SOA-50ms condition (-47ms) than in the SOA-300ms condition (-6ms, F(1,6) = 7.77, p = 0.03). This effect was similar for all sites (premotor/control areas: -34ms, motor area: -18ms, F(1,6) = 0.61, p = 0.47) and no interaction between the two factors was registered (F(1,6) = 0.41, p = 0.55). Interestingly, the pattern of results was different when considering the targets located at an intermediate position. Relative reaction times according to the no-TMS condition was –20ms and there was an interaction between the site and the TMS conditions (F(1,6) = 8.44, p = 0.02). This interaction was explained by the fact that relative reaction times when stimulating the premotor/control area (-59ms) was significantly greater than when stimulating the motor area at SOA-50ms (0.3ms, t(6) = 4.50, p<0.01) but not at SOA-300ms (-13ms and -7ms, respectively, t(6) = 0.39, p = 0.70). Finally, reaction times were greater in the SOA-50ms than in the SOA-300ms conditions for the premotor/control areas (t(6) = 3.52, p = 0.01) but not for the motor area (t(6) = 0.59, p = 0.57). Consequently, the lower facilitation effect of the TMS when applied on the motor area was manifest mainly for those targets located near the boundary of the reachable space.

#### The boundary of what is reachable

**Figure 9:**
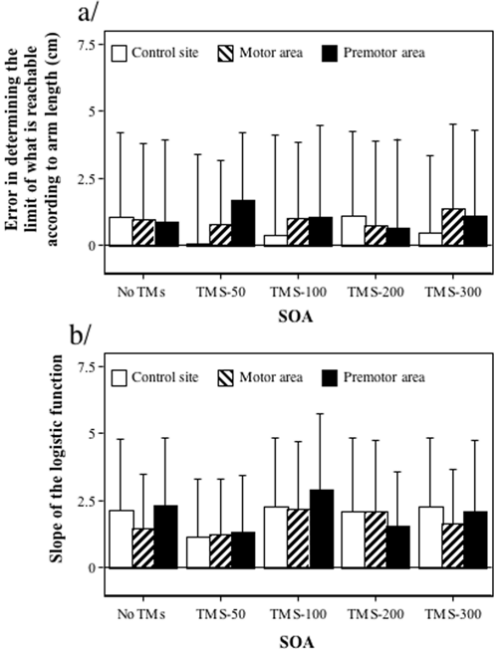
Localisation of the boundary of peripersonal space. (a) Data represent the error in determining the boundary of what is reachable according to arm length (mm) and standard deviations in the perceptual judgement of what is reachable task as a function of the SOA (no TMS, 50ms, 100ms, 200ms, 300ms) and the site (occipito-temporal complex, motor cortex, premotor cortex) where TMS was delivered. (b). Data represent the slope of the logistic function and standard deviations as a function of the SOA and the stimulated site.

Overall, the boundary of what is reachable slightly overestimated arm length (0.88cm) but was not modified by the brain stimulated site (F(2,12) = 0.82, p = 0.45) nor by the SOA (F(4,24) = 0.062, p = 0.99, see Figure 9a). There was also no interaction between the two factors (F(8,48) = 1.26, p = 0.30). Similarly, the slope of the logistic function representing an index of difficulty for the decision task was on average 1.86 and did not vary across the different TMS conditions (F(4,24) = 1.152, p = 0.36) or the different sites (F(2,12) = 0.83, p = 0.45, see Figure 9b). There was also an absence of interaction between the two factors (F(8,48) = 0.25, p = 0.95). Then, the boundary of what is reachable was determined according to arm length and the transient inhibition of the motor system, which affected reaction times, did not modify the decision criteria. The slight overestimation reported here is in agreement with that performance classically observed when the decision task is performed in total darkness.

## Discussion

The aim of the present study was to evaluate the contribution of brain motor areas to the perceptual judgement of whether or not a visual object is reachable, without performing actual movements towards the object. In another condition, a perceptual right-left decision task was administrated as a control task. Our assumption was that the perceptual judgement of whether a visual object is reachable or not required the participation of motor representations, but not the right-left decision task. The method we used was to apply a transient perturbation in the form of a TMS pulse over the motor and premotor cortices when performing the perceptual judgement of what is reachable or the right-left decision task. A control site, the temporo-occipital area, was also used to test the effect of inhibiting brain activity that is far apart from the motor system. It is well known that TMS single pulse has the effect of inhibiting cortical activity during a very short period of time (10–50ms) on a very delimited cortical region (few millimetres) and is considered as similar to a transient virtual cortical lesion [34]. We also analysed the consequences of applying TMS on the same brain regions while performing an actual grasping movement, though the stimulation remained under motor threshold.

First, the results showed a temporal facilitation effect in trials where stimulation was applied, and this was the case in all tasks when stimulating any of the brain sites considered. The magnitude of the facilitation effect was dependent on the SOA between stimulus presentation and TMS onset. Indeed, compared to the no-TMS condition, reaction times were systematically the shortest in SOA-50ms condition (reachability judgement task: -48ms, right-left decision task: -68ms, grasping task: -85ms) and increased progressively up to 200ms-SOA for the reachability judgement task (-2ms) and the grasping task (-21ms), and up to 300ms-SOA for the right-left decision task (-7ms). Such a facilitation effect of the TMS depending on the SOA is in agreement with previous studies that have reported a similar finding when using TMS at subthreshold intensity [37]–[39]. The decrease in reaction times that we observed cannot be attributed to the inhibition of a particular brain area when performing either task. Indeed, the effect was present when stimulating any of the investigated three brain regions. Rather, the general facilitation effect suggests improved processing of the visual target when provided together with other sensory stimulations. The facilitation effect could indeed be associated with the detection of temporally congruent signals at the time the visual target was presented. As reported by the participants, magnetic stimulation was consciously detected through cutaneous conduction on the scalp and/or associated auditory signal. The lack of congruent signals in the no-TMS condition can thus be a possible explanation for the facilitation effect observed when TMS was delivered. The fact that the facilitation effect gradually disappeared when the SOA increased is in agreement with this interpretation and indicates that an external cue presented around the time the target was available improved mainly the early stage of visuo-spatial processing. This result is also in line with the finding that responses to multimodal stimuli combination are faster than responses to unimodal stimulus [50], [51]. In the past, it has been suggested that a combination of visual, auditory, and tactile information determines an inter-sensory facilitation improving time processing [40], [52]. This multisensory facilitation effect was explained in terms of a coactivation mechanism that combines activations from the different modalities to jointly trigger a response [52]. Thus, faster reaction times in the present study when providing TMS pulse close in time to target presentation can be interpreted as improved processing of the visual target in the presence of congruent sensory signals (visual, auditory and tactile) instead of a single one (visual).

The reachability judgement task included however more elaborated processing than the right-left decision task and the grasping task, as suggested by the different reaction times in the no-TMS condition (573ms, 404ms, 431ms respectively). Furthermore, the facilitation effect observed when the TMS was applied was significantly reduced when stimulating the motor cortex, for all SOAs (-34ms on average). The decrease in the facilitation effect in the reachability judgement task might thus be due to the less detectable TMS-related signals when delivered on the motor cortex instead of the premotor cortex or even the control site. However, this interpretation does not fit with the strong sensory experience reported by the participants; it does not explain either the effect of the TMS on that site in the right-left decision task and the grasping task. It also hardly fits with the finding of a reduction in the facilitation effect when considering targets located near the boundary of peripersonal space. Indeed, for stimuli located clearly near or far according to the boundary of peripersonal space, the facilitation effect of the TMS was similar whatever the stimulated site. Consequently, in line with previous studies that have used TMS, the increase in reaction times would rather suggest a disruption of perceptual or cognitive processing, which would occur at that time and would involve the stimulated cortical area [34].

Since premotor cortex has been found in the past to be part of the neural network that is activated during actual motor production, motor imagery and motor cognition [30], [Bibr pone.0002862-Hanakawa2] [53 see 29 for a discussion], it is quite surprising in our experiment that TMS on the premotor cortex did not provide similar effects to that observed when stimulating the motor area. Two explanations can account for the lack of specific effect when providing TMS on the premotor cortex. First, it is possible that the stimulation was not accurately delivered on the premotor site considering the grasping movement employed. Indeed, to define the premotor cortex, we used an empirical method based on scalp coordinates [35], which could have limited the spatial accuracy of TMS position. A more theoretical account could be that the premotor cortex is mainly involved in the selection of movement and less during its execution. In particular Schluter et al. [35] have shown that stimulation of premotor cortex disrupts the early stage of movement selection, whereas stimulating the motor cortex disrupts the movements at a later stage of execution. Because we used a stereotypical motor response varying merely according to the distance of the target, it is thus possible that our movement context limited the computational aspect of response selection, the task requiring subjects to mainly adapt the amplitude of their response.

However, in the present study, the specific effect of TMS when delivered on the motor area strongly suggests that the judgement of what is reachable does not rely on pure visual processing. On the contrary, the motor cortical area appears to contribute to the conscisous judgement of what is reachable, suggesting a motor representation-based processing of visual information. Thus, our claim is that to determine whether an object is reachable or not requires the combination of perceptual information about object location with motor representations about possible movements towards that object. In line with Jeannerod [54], the underlying principle for identifying and selecting reachable objects might thus include a covert action that enables the prediction and the anticipation of the sensory consequences of self-generated movements through an internal predictive model [23], [24], [28]. Simulating an action and anticipating its sensory consequences would provide the means to optimise motor control but also to specify what is reachable in the peripersonal space. However, because the specific effect of TMS when stimulating the motor cortex was absent for very near and very far targets and in fact, it concerned mainly targets close to the boundary of reachable space, one must consider the possibility that action simulation would be required mainly when the determination of what is reachable becomes ambiguous. This idea is in agreement with a previous study that showed that reaction times for the perceptual judgement increase substantially for targets located near the boundary of what is reachable, suggesting a specific process for those particular targets [55]. Similarly, our results indicate that TMS on the motor cortex reduced the facilitation effect on reaction times mainly for stimuli located at the boundary of what is reachable. It is thus tempting to assume that especially for stimuli located at an ambiguous location according to the peripersonal space, a covert action is required in order to code the spatial stimulus in motor terms and improve the decision process [26]. It is in fact conceivable that for estimating whether a visual object is reachable or not in the very near or very far space, covert motor activity is not required since information about the geometry of surface layout and objects as revealed in optical and ocular-motor variables would be sufficient-in principle-to dissociate what is reachable from what is not reachable, by relying on those mechanisms that are different from those used for the motor simulation process. In contrast, for targets located near the boundary of peripersonal space, the motor cortex revealed to be part of the neural network that contributes to the perceptual judgement of what is reachable. Interestingly, the fact that stimulating the occipito-temporal area did not influence the perceptual tasks indicates that, at least in our study, this area may not participate in the conscious processing of objects' spatial characteristics, as this could have been expected following the perception-action theory [9].

Though surprising, the TMS did not influence the critical distance at which the boundary of what is reachable was perceived. Participants slightly overestimated the area including all reachable objects (+0.88cm) but this overestimation was not different depending on the stimulated site. This observation is however consistent with the finding in previous studies of a consistent bias towards overestimating true arm length when judging what is reachable [15]–[19]. As discussed in the introduction, this overestimation could result from everyday experience of reaching, which naturally requires multiple skeletal degrees of freedom, whereas they are generally tested in restricted postural situations that prevent natural body movements [17]–[19]. It could also result from the limited information provided by the visual system when the judgement of what is reachable is tested in poor visual condition [20].

Finally, we did not find any significant effect of TMS on the kinematics of the grasping action. This indicates that TMS did not modify substantially motor execution, in agreement with previous study [37]. However, in this respect other data have suggested that TMS can produce inhibition of subsequent motor evoked potentials [56] with a decrease of ongoing EMG activity at target muscles [57]–[59]. Complementary studies would be necessary to reconcile these controversial data.

To conclude, because of the constant variation of postural constraints, the boundary of peripersonal space would vary accordingly and judging what is reachable would require combining visual information with representation of the body newly constructed moment by moment [23]. This study provides the first evidence that motor representations contribute in the specification of the boundary of peripersonal space. Future lines of research could evaluate whether the specific modular effects that were observed in this study when stimulating the motor area could be sustained through time, for instance by using repetitive TMS before performing the reachability judgment task [e.g. 60].
